# Disruption of Genes Encoding Putative Zwitterionic Capsular Polysaccharides of Diverse Intestinal Bacteroides Reduces the Induction of Host Anti-Inflammatory Factors

**DOI:** 10.1007/s00248-022-02037-1

**Published:** 2022-05-21

**Authors:** Kathleen L. Arnolds, Eiko Yamada, C. Preston Neff, Jennifer M. Schneider, Brent E. Palmer, Catherine A. Lozupone

**Affiliations:** 1 Department of Immunology and Microbiology, University of Colorado Anschutz, Aurora, CO USA; 2 Department of Medicine, University of Colorado Anschutz, Aurora, CO USA

**Keywords:** Zwitterionic capsular polysaccharides, ZPS, PSA, Treg, IL-10

## Abstract

Bacterial zwitterionic capsular polysaccharides (ZPS), such as polysaccharide A (PSA) of the intestinal commensal *Bacteroides fragilis*, have been shown to modulate T cells, including inducing anti-inflammatory IL-10-secreting T regulatory cells (Tregs). We previously used a genomic screen to identify diverse host-associated bacteria with the predicted genetic capacity to produce ZPSs related to PSA of *B. fragilis* and hypothesized that genetic disruption (KO) of a key functional gene within these operons would reduce the anti-inflammatory activity of these bacteria. We found that ZPS-KO bacteria in two common gut commensals, *Bacteroides uniformis* and *Bacteroides cellulosilyticus*, had a reduced ability to induce Tregs and IL-10 in stimulations of human peripheral blood mononuclear cells (PBMCs). Additionally, we found that macrophage stimulated with either wildtype *B. fragilis* or *B. uniformis* produced significantly more IL-10 than KOs, indicating a potentially novel function of ZPS of shifting the cytokine response in macrophages to a more anti-inflammatory state. These findings support the hypothesis that these related ZPS may represent a shared strategy to modulate host immune responses.

**Supplementary Information:**

The online version contains supplementary material available at 10.1007/s00248-022-02037-1.

## Introduction

Millenia of interplay between the microbiota and host immune responses has shaped the evolution of each, leading to dynamic relationships between microbes and man. While it is clear these interactions are important to multiple aspects of human physiology, in many cases, the mechanisms driving microbial influence of the host remain to be described. To gain insight into these relationships, we can directly assess the interface between microbes and host factors. In the gut, many of these interactions occur at the mucosa overlaying the intestinal epithelium and are mediated by interaction between capsular factors of bacteria and host immune receptors. Here, we investigate the role of bacterial capsular polysaccharides, specifically zwitterionic polysaccharides (ZPS), on the host immune system.

ZPSs contain alternating positive and negatively charged residues, unlike most capsular polysaccharides which are negatively charged. In some cases, the positive charge is conferred by an acetamido-amino-2,4,6-trideoxygalactose amino sugar (AATGal-ZPS) [1]. The most well studied AATGal-ZPS is polysaccharide A (PSA) from *Bacteroides fragilis*, a common gut microbe [2]. PSA has been shown to induce FoxP3 + CD4 + T regulatory cells (Tregs) and interleukin 10 (IL-10) secretion [3]. Evidence supports that T cell activation by ZPS is mediated by activation of antigen-presenting cells (APCs) via Toll-like receptor 2 (TLR2), which then directly interact with T cells in a MHC class II-dependent manner [3–6]. PSA can rebalance biased Th17/Treg levels to mitigate pathologic inflammation [7] and has been shown to be protective in a mouse colitis model [8], suggesting therapeutic potential. However, *B. fragilis* is one of the most pathogenic species in the *Bacteroides* genus, for instance, being the leading cause of intra-abdominal abscess, which it promotes in a PSA-dependent manner [9].

Although PSA of *B. fragilis* represents the most well-studied AATGal-ZPS, structurally and functionally related immunomodulatory AATGal-ZPS occur in other organisms such as SP1 of *Streptococcus pneumoniae* [10, 11]. Similarly, other AATGal-ZPS have been described that are carried by different strains of *B. fragilis*, such as PSA2 [11]. Using a genomic screen, our lab had previously identified hundreds of bacterial genomes from dozens of species in diverse bacterial orders that contain putative AATGal-ZPS operons with highly conserved genes from the PSA operon of *B. fragilis* [11]. Briefly, our lab screened > 8000 genomes for orthologues to the *wcfR* gene that encodes AATGal synthase and additionally determined whether other conserved elements of the ZPS operon in *B. fragilis* were nearby in the genome. The operons identified by this screen had diverse gene content and included PSA2 of *B. fragilis* and SP1 of *Streptococcus pneumoniae* [10, 11]*.* We found that stimulation of PBMCs with lysates from bacteria we predict to produce a ZPS induced higher levels of Tregs and increased levels of the cytokine IL-10 compared to their phylogenetic relatives that do not encode a ZPS [11]. However, the comparison between putative ZPS producers and closely related non-producers is complicated by the fact that these phylogenetic relatives differ in many other genes besides the putative ZPS operon [11]. In our prior work, genetic disruption (KO) of one of 2 predicted ZPS in the genome of the intestinal commensal *Bacteroides cellulosilyticus* resulted in reduced IL-10 production and Treg numbers after stimulation of both PBMC and antigen presentation to purified naïve CD4 + T cells [11].

Here, we expand this work to show loss of these same activities upon genetic disruption of the second ZPS of *B. cellulosilyticus* and of the predicted ZPS operon of *Bacteroides uniformis*. We focused on these bacteria because we and others have found them to be associated with anti-inflammatory immune responses [11–16]. While we saw that our KOs indeed had a reduction in Tregs, we also uncovered anti-inflammatory effects on macrophage activity, further expanding our knowledge of impacts of ZPS on host immunity.

## Results

### Developing ZPS-Knockout Strains

**Fig. 1:**
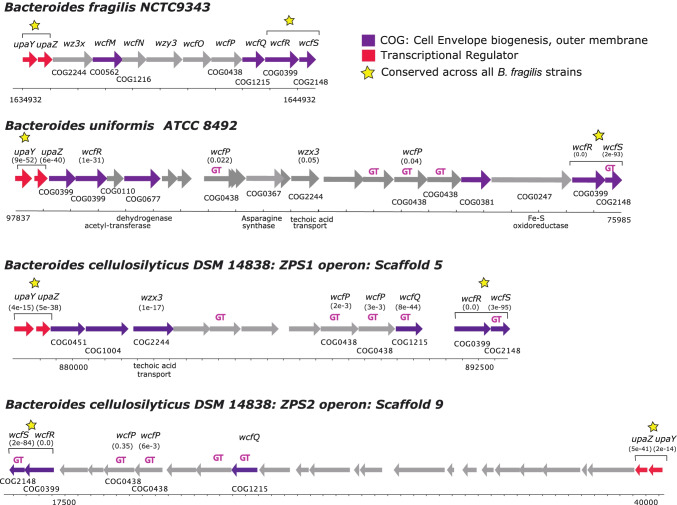
Predicted AATGal operons of *B. fragilis*, *B. cellulosilyticus*, and *B. uniformis*. Genes labeled GT represent glycosyltransferases. Homologs to genes in the PSA operon of *B. fragilis* are noted with their e-value in parentheses. The yellow stars indicate genes that were conserved in the AATGal-ZPS operons of 50 evaluated strains of *B. fragilis* in Coyne et al. [41]

Using an insertional mutagenesis knock-out strategy, we developed a single ZPS KO strain of *B. uniformis* ATCC8492 and two unique KO strains of *B. cellulosilyticus* DSM 14,838. *B. uniformis* ATCC8492 has a single putative ZPS operon, containing close homologs to several genes in the PSA operon of *B. fragilis*, including the *wcfR* gene for AATGal synthase, *wcfS* and *wcfP* glycosyltransferase (GT) encoding genes, the *wzx3* teichoic acid transport gene, and the transcriptional regulators *upaY* and *upaZ* (Fig. 1). The *B. uniformis* ZPS operon actually contains two different *wcfR* homologs, but only the copy adjacent to *wcfS* reached our previously determined similarity threshold [11] for identifying putative orthologues of the wcfR gene. *B. cellulosilyticus* DSM 14,838 has two unique putative AATGal-ZPS operons each with its own *wcfR* orthologue; one is encoded on Scaffold 5 (ZPS1) and one on Scaffold 9 (ZPS2) [11]. We developed two unique KO strains in which one operon had been disrupted and the other remained intact, *B. cellulosilyticus* ΔZPS1 and *B. cellulosilyticus* ΔZPS2. The operon encoding ZPS1 is simpler than the operon encoding ZPS2 and shows greater similarity in gene content to PSA, sharing homologs to *wcfR*, the GTs *wcfS*, *wcfQ*, and *wcfP* and to the *wzx3* teichoic acid transport gene and the *upaY* and *upaZ* transcriptional regulators (Fig. 1) [11]. ZPS2 also has homologs to *wcfR* and the *wcfS*, *wcfQ*, and *wcfP* GTs and the *upaY* and *upaZ* transcriptional regulators but lacks a *wzx3* gene homolog and has many more unique genes (Fig. 1).

To develop the KO strains, *wcfR*, the gene in the ZPS operon that encodes AATGal synthase, was targeted. We targeted this gene because in principle, the disruption of *wcfR* would result in the abrogation of ZPS activity by impairing the assembly of the sugar subunits with alternating charges. We confirmed basal expression of *wcfR* under our culture conditions using primers that targeted conserved regions of the *wcfR* gene (Supplemental Fig. 1; Supplemental Table 1) and that both ZPS1 and ZPS2 were expressed in WT *B. cellulosilyticus* DSM14838 using primers specific to the *wcfR* gene of each operon (data not shown). *WcfR* was disrupted via insertional mutagenesis using the pKNOCK-bla-ermGb plasmid [17]. Genomic disruption of *wcfR* was confirmed with PCR using a forward primer specific to the *wcfR* gene and a reverse primer specific to the plasmid, RT-PCR was used to validate a loss of *wcfR* message, and strain identity was confirmed by sequencing the 16S rRNA gene (Supplemental Fig. 2). By PCR confirmation of both genomic DNA and mRNA, we confirmed that the *wcfR* gene of ZPS1 and not ZPS2 of *B. cellulosilyticus* was disrupted in ΔZPS1 and vice versa and that the single *wcfR* gene of *B. uniformis* was disrupted (Supplemental Fig. 2).

**Fig. 2:**
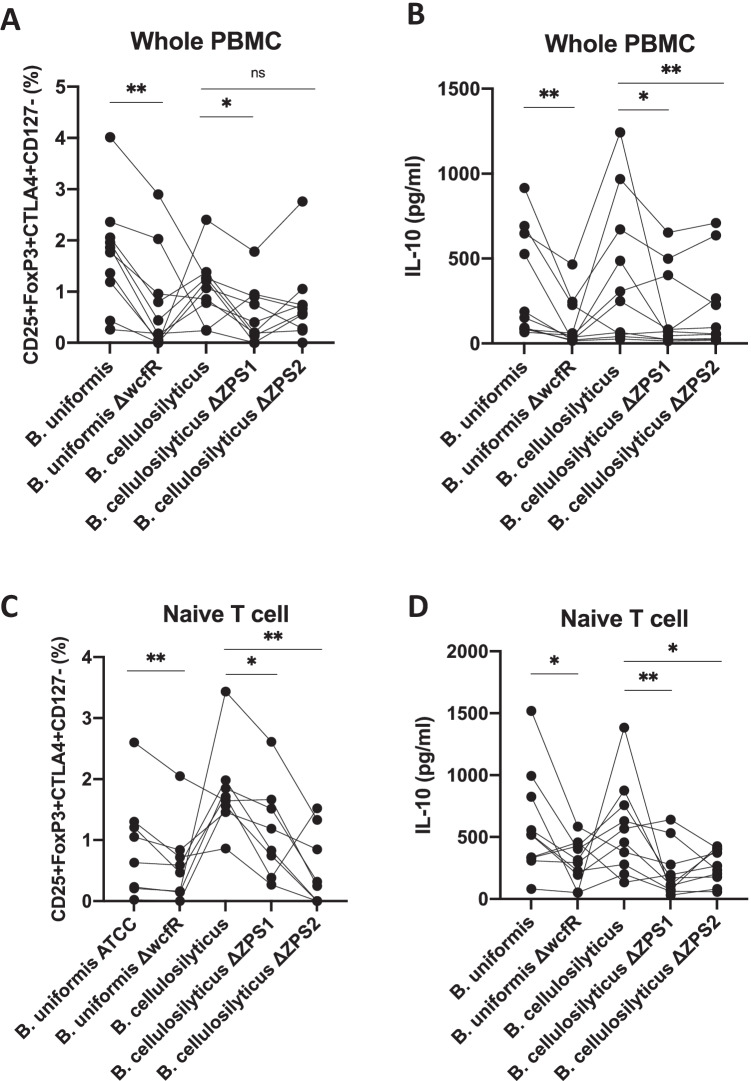
Stimulation with wild-type ZPS producers induce more Tregs and IL-10 from PBMC than stimulation with KO strains. **A** and **C** represent the proportion of CD4 + T cells that are CD25 + FoxP3 + CTLA4 + CD127 (Tregs) after subtraction of the % stimulated by culture media controls. **B** and **D** represent the levels of IL-10 in the supernatant determined by ELISA after 3 days of culture with bacterial lysates; No subtraction from culture media controls was applied here because control (*n* = 5; no bacteria) stimulations had IL-10 levels below the limit of detection. **A** and **B** represent stimulation of whole PBMC. **C** and **D** represent co-culture of purified naïve T cells with CD14 + monocytes that had been stimulated with bacterial lysates. Each bacterial lysate was tested across PBMCs from the same set of individuals, with different sets of 10 PMBCs used for **A**, **B** and **C**, **D**. Result from the same individual is joined with a line. **C** shows only 8 data points since 2 of the 10 PBMCs had no response from any lysate. Statistical significance between wildtype and KO was determined using the paired Wilcoxon test. *p < 0.05, **p < 0.01. A representative staining for CD25 + FoxP3 + CTLA4 + CD127 − Tregs in human PBMC and naïve T cells is shown in Supplemental Fig. 3

### ZPS-KO Strains of B. cellulosilyticus and B. uniformis Elicit Lower Levels of Tregs and IL-10 in PBMCs

To assess the impact of disruption of *wcfR* gene homologs on the immune-modulatory capacity of *B. cellulosilyticus* and *B. uniformis*, we first analyzed the proportion of CD4 + T cells that were CD4 + CD25 + FoxP3 + CTLA4 + CD127 − Tregs and the level of the cytokine IL-10 produced by PBMC after stimulation with either the WT strains or the KOs. A reduced proportion of Tregs was observed in the PBMC cultured with lysate from *B. uniformis* ΔwcfR (*p* = 0.002), *B. cellulosilyticus* ΔZPS1 (*p* = 0.037), but not *B. cellulosilyticus* ΔZPS2 (*p* = 0.106) versus those cultured with WT strains (Fig. 2A). Similarly, culture with lysate from KOs resulted in lower levels of IL-10 versus those cultured with lysate from WT strains for *B. uniformis* ΔwcfR (*p* = 0.002), *B. cellulosilyticus* ΔZPS1 (*p* = 0.049), and *B. cellulosilyticus* ΔZPS2 (*p* = 0.004) (Fig. 2B).

Induction of Tregs and IL-10 by PSA from *B. fragilis* has been shown to be mediated in part via antigen-presenting cell (APC) presentation to naïve CD4 + T cells, a phenotype shared by other putative ZPS producers [11]. When we isolated APCs and cultured them with lysate from *B. uniformis* WT, *B. uniformis* ΔwcfR, and *B. cellulosilyticus* WT, ΔZPS1, or ΔZPS2 and then co-cultured them with purified naïve CD4 + T cells, a significant reduction in the proportion of Tregs was observed for *B. uniformis* ΔwcfR (*p* = 0.008), *B. cellulosilyticus* ΔZPS1 (*p* = 0.016), and *B. cellulosilyticus* ΔZPS2 (*p* = 0.008) compared to WT bacteria (Fig. 2C), with a greater reduction in the percentage of Tregs induced by *B. cellulosilyticus* ΔZPS2 versus ΔZPS1. All KO bacteria also induced less IL-10 compared to WT bacteria in this assay (*B. uniformis* ΔwcfR (*p* = 0.037), *B. cellulosilyticus* ΔZPS1 (*p* = 0.010), and *B. cellulosilyticus* ΔZPS2 (*p* = 0.049)), with a greater reduction in the amount of IL-10 in *B. cellulosilyticus* ΔZPS1 versus ΔZPS2 (Fig. 2D). Since these assays still contain the APCs and other types of CD4 + T cells, this IL-10 is not necessarily from the Tregs. Taken together, these results suggest that ZPS of *B. cellulosilyticus* and *B. uniformis* both influences IL-10 and Treg induction in complex cell populations present in PBMC and also specifically affects the differentiation of naïve T cells to Tregs. However, *B. cellulosilyticus* ΔZPS2 induced significantly lower levels of IL-10 compared to WT despite not inducing a significantly lower proportion of Tregs. Similarly, *B. cellulosilyticus* ΔZPS2 had a greater reduction in Treg differentiation compared to ΔZPS1 in naïve T cell assays, but a smaller reduction in IL-10. This indicates that the observed changes in IL-10 are not fully explained by differences in Treg numbers and may indicate other immune cell types are involved, such as other types of T cells, Natural killer (NK) cells, monocytes, or dendritic cells, which are all present in PBMC and capable of producing IL-10 in specific conditions [18–21].

### IL-10 Production from Macrophages Is Reduced in Response to KO Strains of B. fragilis and B. uniformis Versus WT

**Fig. 3:**
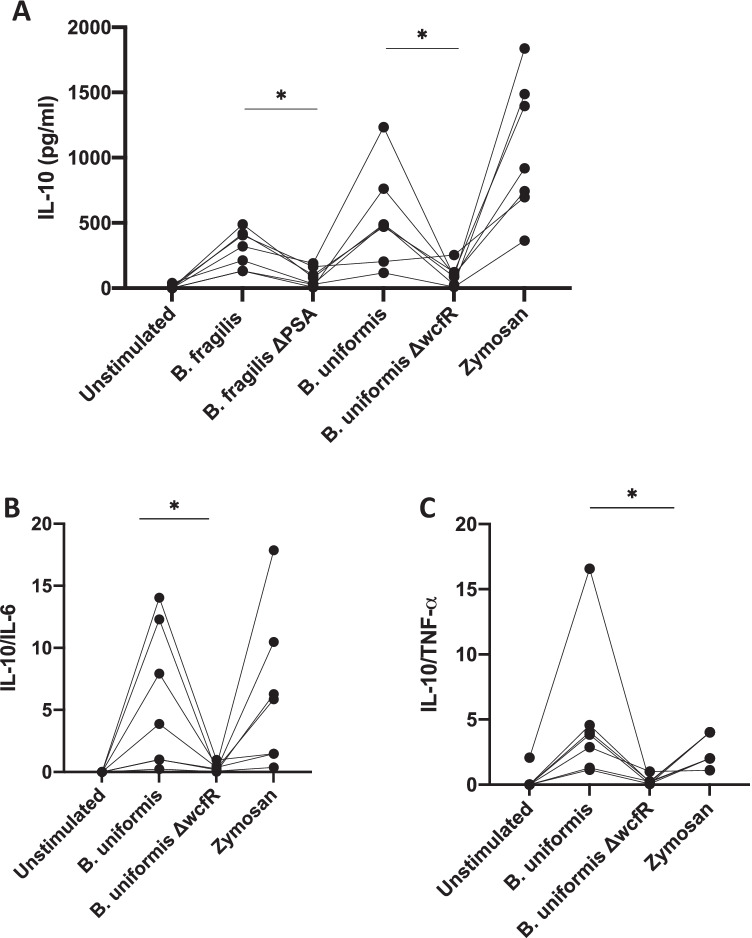
Stimulation of derived macrophages with *B. fragilis* or *B. uniformis* induces more IL-10 and *B. uniformis* induces greater IL-10/TNF-α, IL-10/IL-6 ratios than stimulation with KO strains. Macrophages were stimulated with heat-killed bacterial lysates for 6 h, supernatant was collected, and ELISA was used to assess IL-10, TNF-α, and IL-6 levels. **A** IL-10 levels, **B** ratios of IL-10/IL-6, and **C** ratios of IL-10/TNF-α. Each bacterial lysate was tested across derived macrophage preps from the same set of individuals (result from same individual is joined with a line). Statistical significance was calculated by the paired Wilcoxon test. Data were the result of *n* = *7* PBMC from different paired individuals. *p < 0.05

Intestinal macrophages are important mediators of immune homeostasis in the gut [22]. To assess the role for macrophages in the response to ZPS producing bacteria, CD14 + cells were isolated from healthy PBMC and differentiated into macrophages using M-CSF (Supplemental Fig. 4) and were then cultured with bacterial lysates. Macrophages cultured with either WT *B. fragilis* or *B. uniformis* produced more IL-10 than those cultured with *B. fragilis* ΔPSA (*p* = 0.016) or *B. uniformis* ΔwcfR (*p* = 0.031) (Fig. 3A).

Although IL-10 is generally a hallmark of an anti-inflammatory response, it can also simply indicate activation; therefore, we also assessed the ratios of IL-10 to TNF-α and IL-10 to Il-6 for *B. uniformis*. Both the IL-10/TNF-α and IL-10/IL-6 ratios were greater in response to *B. uniformis* WT than *B. uniformis* ΔwcfR (*p* = 0.016, *p* = 0.016, respectively), and no difference was seen for IL-6 or TNF-α levels alone (Supplemental Fig. 5), indicating that ZPS may shift the macrophage cytokine response to a more anti-inflammatory state (Fig. 3B, C). Zymosan, a TLR-2 agonist from *Saccharomyces cerevisiae*, was used as a control for macrophage stimulation.

## Discussion

ZPS encoding strains of *B. cellulosilyticus* and *B. uniformis* influenced IL-10 and Treg induction in both complex cell populations present in PBMC and through the differentiation of naïve CD4 + T cells to Tregs upon co-culture with ZPS exposed APCs, indicating similar anti-inflammatory effects to those previously described for *B. fragilis* PSA [11]. In addition, ZPS represent a family of related molecules, and our data support that they have non-identical immune modulatory properties. The loss of anti-inflammatory activities with genetic disruption in all the ZPS operons from our genomic screen tested thus far suggests that this pool of ZPS encoding bacteria is a rich resource for identifying anti-inflammatory bacteria and ZPS molecules for further study. Since *B. fragilis* is one of the most virulent species in the Bacteroides genus [23], the bacteria described here may be better candidates for therapeutic efforts targeting inflammatory disease. Previous studies of both *B. cellulosilyticus* and *B. uniformis* have indicated protective effects in different health contexts. Our previous work showed that probiotic administration of *B. cellulosilyticus* protected from disease TNBS induced colitis in mice and increased Tregs [11]. Further studies conducted using the KO bacterial strains that we generated or purified ZPS would help to elucidate a role of their ZPS in these protective phenotypes.

The presence of two ZPS operons in *B. cellulosilyticus* suggest some degree of redundancy that can obscure the impact of disruption of one gene on bacterial functions. With that said, it is interesting that both *B. cellulosilyticus* ΔZPS1 and ΔZPS2 showed some loss of immune-modulatory function despite having one functionally intact ZPS operon, suggesting additive effects. Our results with the two different *B. cellulosilyticus* KO strains also suggested unique functions of different ZPS. Specifically, *B. cellulosilyticus* ΔZPS2 had a significant loss in the amount of IL-10 induced compared to WT despite not having a significant loss in the amount of induced Tregs. Similarly, *B. cellulosilyticus* ΔZPS2 had less Treg differentiation compared to ΔZPS1 in naïve T cell assays, but a smaller loss of IL-10 production. This might indicate that different ZPS have differential interactions with various cell populations. Indeed, PBMC represents a complex milieu of different cell types capable of producing IL-10 under certain conditions, including NK cells, monocytes, and dendritic cells [20]. In our previous work [11], we used a 24-h intracellular cytokine staining (ICCS) assay to evaluate IL-10 production by CD4 + T cells in stimulations of PBMC with wildtype *B. fragilis* versus *B. fragilis* ΔPSA. We found that wildtype *B. fragilis* stimulations produced a higher fraction of CD4 + IL-10 + cells than *B. fragilis* ΔPSA. However, the majority of these CD4 + IL-10 + cells were not expressing the T reg markers CD25 and FoxP3, supporting that IL-10 levels in our PBMC assays may also be driven by other types of T cells. It would be compelling to further explore variability of levels of IL-10 production and the specific cell types involved using the *B. cellulosilyticus* KO strains, which would allow us to probe how different ZPS in the same genomic background might differentially impact host responses.

This work has also produced a novel finding that genetic disruption of the ZPS operons of *B. fragilis* PSA and the related ZPS of *B. uniformis* resulted in a reduction of IL-10 production from macrophage. *B. uniformis* WT also induced higher IL-10/IL-6 and IL-10/TNF-α ratios from differentiated macrophage than the ZPS KO strain, suggesting that a change in the cytokine milieu from macrophage induction may be an early response to ZPS that could contribute to downstream augmentation of Tregs. IL-10 secreted by macrophages in the lamina propria has been linked with maintenance of FoxP3 expression in Tregs under inflammatory conditions [24]. Macrophages are critical players in both protective inflammatory responses and in maintaining gut immune homeostasis [22], and prior studies have indicated that other microbes can also elicit anti-inflammatory effects via macrophage stimulation by polysaccharides. Specifically, a large polysaccharide produced by *Helicobacter hepaticus* activates intestinal macrophages via TLR2 resulting in an anti-inflammatory response characterized by high IL-10/IL-6 ratios [25]. Both *H. hepaticus* and *B. fragilis* can be highly pathogenic in IL-10 ^−/−^ mice [26, 27], indicating the importance of IL-10 stimulating factors in maintaining homeostasis between microbes and host.

We note that using M-CSF to drive macrophage differentiation does not target a M1 or M2 lineage commitment and may also result in some monocyte-derived dendritic cells; thus, the assay that we employed may be missing some aspects of the mechanism. It would be valuable to investigate the roles of these specific cell subsets and further investigate if longer stimulations with bacterial products would drive the cell lineage one way or the other.

Although our study provides further support regarding the potential anti-inflammatory properties of ZPS encoding bacteria, we do acknowledge that our study has weaknesses. For one, genetic disruption of the *wcfR* gene has the potential to change the expression of other genes in the bacterium as a compensatory mechanism, but we did not evaluate transcriptomic differences in the wildtype versus KO bacteria. We also only validated disruption of *wcfR* by ablation of the gene transcript and not by ZPS molecule loss, which is challenging because these are predicted ZPS based on a genomic screen that have not yet been purified and characterized biochemically. Since purification and biochemical validation of ZPS is challenging and our genomic screen has identified many putative ZPS, experiments demonstrating a loss of anti-inflammatory function with genetic disruption are an important first step to identify the optimal candidates for further in-depth characterization.

The purification and biochemical characterization of the most promising ZPS would allow for further mechanistic experiments to address their promise as treatments for inflammatory disease, as has been conducted for PSA of *B. fragilis* [7, 8, 11, 28]. Assessing the immune responses to purified ZPS would also help to clarify the specific effect of a ZPS alone versus impacts of loss of that ZPS in the context of other microbial components found in the lysates, such as lipopolysaccharide (LPS). While LPS is canonically associated with inducing innate defense responses [29], some *Bacteroides* LPS can also aid in immune evasion or promote anti-inflammatory cytokine responses [30, 31]. Prior studies have shown that the initiation of an anti-inflammatory response by PSA from *B. fragilis* depends on a covalently bonded lipid factor, lipid A [32]. It would be compelling to investigate if a lipid A type structure is similarly required for the ZPS from other *Bacteroides* strains and if our knock-out strategy disrupted the presentation of this molecule.

The rapid expansion of microbiome studies has inspired great interest in poorly characterized bacterial strains, and while this presents great potential, it also brings unique challenges as the biology of many of these bacteria remain incompletely understood. The work presented here contributes to the foundation for more focused studies to explore how these bacteria influence our health and how we can harness those abilities to prevent or mitigate inflammatory diseases.

## Methods

### Growth of Isolates and Lysate Preparation

Isolates were purchased from the ATCC or DSMZ and grown in rich media (Mega Media [33]). When robust culture growth was observed (typically after 18–36 h depending on the bacteria), liquid cultures were centrifuged at 2500 rpm for 10 min. Supernatant was removed, and the pellet was resuspended in PBS and frozen at − 80 °C. Resuspended bacteria were subjected to freeze/thaw and heat killing at 65 °C for 30 min before being used in immune stimulations. To determine whether the *wcfR* gene was being expressed under the growth conditions employed, we designed primers that specifically amplified the *wcfR* gene using PrimerProspector software [34]. We developed a primer set that exclusively amplified most *wcfR* genes in the Bacteroides genus and verified the presence of *wcfR* mRNA in cultures of WT *B. cellulosilyticus*, *B. fragilis*, and *B. uniformis*. As negative controls, we attempted to amplify from *B. fragilis* ΔPSA and *B. intestinalis*. The sequences of this primer set are given in Supplemental Table 1. Since this primer set amplified *wcrR* from both ZPS1 and ZPS2 of *B. cellulosilyticus*, we also designed primers that specifically amplified the *wcrR* gene of each ZPS so that we could ensure that both ZPS operons were being expressed (Supplemental Table 1). RNA was extracted from bacterial cultures using the PureLink RNA Mini Kit from Life technologies. RNA was reverse transcribed to cDNA using ThermoScript RT-PCR system from Invitrogen. Standard PCR was then performed on cDNA to amplify *wcfR* in various bacterial strains.

### Generating wcfR Knockouts

The KO strains were developed using the pKNOCK-bla-ermGb vector to disrupt the *wcfR* gene via targeted insertional mutagenesis as previously described [35]. Briefly, a ~ 200 base pair internal fragment of the target gene, *wcfR*, was first amplified using the genomic DNA of the targeted strain (*B. uniformis* or *B. cellulosilyticus*) and the strain-specific insert primers (listed in Supplementary Table 1). The resulting amplicon was digested and ligated into the pKNOCK-bla-ermGb vector, which was then electroporated into *Escherichia coli* S17-1 lambda pir. Conjugation and plasmid transfer into *B. uniformis* and *B. cellulosilyticus* from *E. coli* S17-1 lambda pir was then initiated by co-culture of the two bacteria on BHI blood agar plates under aerobic conditions for 36 h. As the *E. coli* growth becomes dense, the conditions become more anaerobic facilitating conjugation and growth of transformed *Bacteroides*. Disruption of the *wcfR* gene occurs via insertion of the pKNOCK plasmid by homologous recombination. Insertion of the pKNOCK plasmid confers ampicillin and erythromycin resistance, which allowed for selection of *wcfR* KOs by growth in media containing 50 μg/mL of erythromycin. Successful disruption of *wcfR* was confirmed by attempting to amplify with primers within the *wcfR* gene that flank the insertion site using the primers listed in S. Table 1. Because *B. cellulosilyticus* DSM14848 has 2 highly related copies of *wcfR*, we selected primers that were specific to one copy and not the other. We also confirmed that the plasmid had integrated into the *wcfR* gene by amplification of genomic DNA with a forward primer specific to the highly conserved region of *wcfR* and a reverse primer specific to the plasmid confirmed insertion of pKNOCK-bla-Erm-gB (primers 495f and pKNOCK_ermGb in Supplementary Table 1). Finally, we also confirmed that the mRNA of only one copy of *wcfR* was being made using PCR with *wcfR* targeted primers to each individual *B. cellulosilyticus* scaffold (Supplemental Table 1) and by Sanger sequencing (data not shown). All PCR was conducted on a BioRad C1000 thermocycler using MyTaq (Bioline); the following PCR program was run for 30 cycles; 94 °C 3 min, (94 °C 45 s, 50 °C 60 s, 72 °C 90 s) × 29, 72 °C 10 min.

### PBMC and Naïve T Cell Assays

Informed consent was obtained, and the study protocol was approved by the Colorado Multiple Institutional Review Board (COMIRB #14–1595, #17–0348). Human PBMCs were isolated by Ficoll density gradient centrifugation as previously described [36–38] from the blood of 10 individuals. 1 × 10^6^ PBMCs were cultured with 10 μg freeze killed bacterial lysate in 500 μl media in 48-well flat-bottom plates for 3 days at 37 °C. Cultures were performed in the presence of 1% of streptomycin and penicillin and in aerobic conditions, and no bacterial growth was observed in the cell cultures. Unlike in the assays conducted by Kreisman and Cobb [39] in human PBMC, our stimulations were conducted in the absence of exogenous IL-2; the IL-2 receptor CD25 is upregulated in the presence of IL-2, which could compromise our Treg staining.

To quantify cytokine secretion after 3 days of culture with bacterial lysates, supernatant was collected and subjected to IL-10 Human ELISA kit (Thermo Fisher, Waltham, MA, USA) following the manufacturer’s instructions. ELISA plates were coated overnight with the specific capture antibody and then washed and blocked with assay diluent for 30 minutes. One hundred microliters of supernatant from three-day culture with bacterial lysate was evaluated for cytokine as per instructions supplied in the kit. To enumerate Tregs, cells were washed with staining buffer containing PBS, 2% BSA, 2 mM EDTA, and 0.09% NaN3, and surface staining was performed with BV605-labeled anti-CD3 antibody (BioLegend, San Diego, CA, USA), PerCP/Cy5.5-labeled anti-CD4 (BioLegend), BV421-labeled anti-CD8 antibody (BioLegend), APC-Cy7-labeled anti-CD25 (BioLegend), and FITC-labeled anti-CD127 (BioLegend). Intracellular staining for PE-labeled anti-FoxP3 antibody (eBioscience, San Diego, CA, USA), APC-labeled anti-CTLA-4 antibody (BD Biosciences, Franklin Lakes, NJ, USA), was performed using the FoxP3 staining buffer set (eBioscience). Cells were then subjected to surface staining and fixed as above and permeabilized using Fix and Perm (Life Technologies, Waltham, MA, USA) for 30 min. Cells were washed 2 times and enumerated with a LSR II (BD Biosciences), and data were analyzed using FlowJo software (Treestar, Ashland, OR, USA). To rule out if induced IL-10 production and Tregs were due to restimulation of memory T cells, 5 × 10^5^ CD14 + monocytes were isolated by magnetic bead selection (Miltenyi Biotec, Auburn, CA, USA), plated in 500 μl media in 48-well flat-bottom plates, stimulated with bacterial lysate for 4 hours, and washed twice with PBS. 7 × 10^5^ naïve T cells were negatively selected by magnetic beads (Miltenyi Biotec) and were added to the bacterial stimulated APCs. After 3 days, the supernatant was collected and subjected to IL-10 ELISA, and cells were enumerated for Tregs as above.

### Macrophage Protocol

CD14 + monocytes were positively selected out of 7 different PBMCs using CD14 Microbeads, human (Miltenyi Biotech). Purified CD14 + cells were plated at a concentration of 1 × 10^6^/1 mL in complete RPMI (Thermo Fisher) supplemented with 20 ng/ml M-CSF. RPMI contains 10% heat inactivated human serum. 1% concentration of penicillin and streptomycin was additionally added. Cells were incubated for 7 days and were washed and given fresh media every 3 days. Differentiation was monitored by light microscopy and flow cytometry as described below. Upon differentiation, cells were harvested with EDTA-trypsin and transferred into a 24-well plate at a concentration of 500 K cells/ml. Cells were then stimulated for 6 h with heat killed bacterial lysate or Zymosan (10 μg/mL, InvivoGen). Supernatants were harvested and directly analyzed by ELISA IL-10, IL-6, and TNF-α (ELISA kits, Thermo Fisher). Zymosan has been shown to activate the CREB pathway which can result in the induction of genes associated with a regulatory or anti-inflammatory macrophage response [40]. CD14 mean fluorescence intensity (MFI) was evaluated with flow cytometry since CD14 is shed from monocytes upon differentiation to macrophage. Specifically, cells were incubated with anti-human CD14 mAB for 30 minutes at 4 °C in the dark. Cells were then washed and fixed and analyzed on a BD FACS CANTO. Data was analyzed using FlowJo software.

### Statistical Analysis for Differences in Immune Cell Populations/Cytokines

Statistical analyses assessing differences in immune cell populations and cytokines in stimulations with wildtype versus KO bacteria were performed using GraphPad Prism (GraphPad, San Diego, CA, USA). Values from controls (culture media without bacteria) were subtracted from plotted values when above the limit of detection. Paired Wilcoxon tests were used for pairwise comparisons and paired Friedman tests when more than 2 groups were being compared. A *p* value of < 0.05 was considered statistically significant.

## Supplementary Information

Below is the link to the electronic supplementary material.

Supplementary file1 (DOCX 2938 KB)

## Data Availability

The KO strains generated in this study will be shared upon reasonable request.
