# Supplementary material for: Disruption of Genes Encoding Putative Zwitterionic Capsular Polysaccharides of Diverse Intestinal Bacteroides Reduces the Induction of Host Anti-Inflammatory Factors
Source: Microb Ecol. 2022 May 21;85(4):1620–9. doi: 10.1007/s00248-022-02037-1 (PMC10167101; doi:10.1007/s00248-022-02037-1)
Supplement: Supplementary file 1 — Supplementary file1 (DOCX 2938 KB) [file 248_2022_2037_MOESM1_ESM.docx]

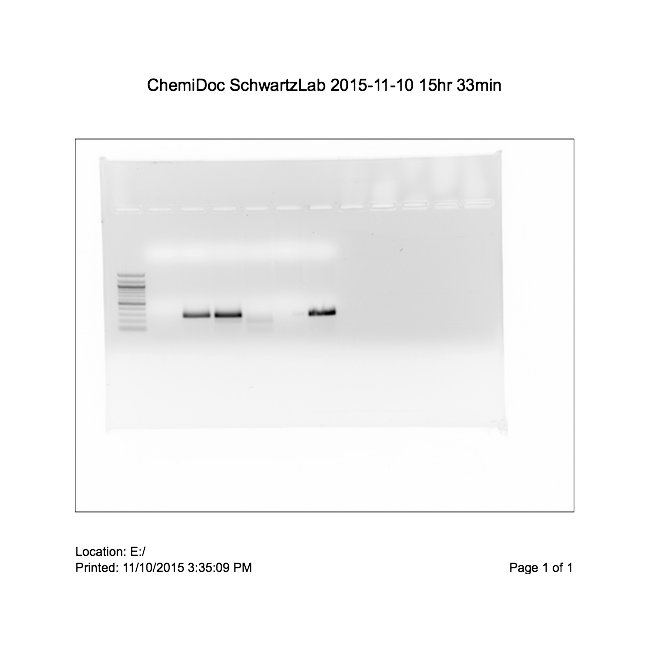


dH20

*B. cellulosilyticus*

*B. fragilis*

*B. fragilis Δ PSA*

*B. intestinalis*

*B. uniformis*

**Supplemental Fig. 1 Confirming *wcfR* expression in putative ZPS producers.**

Amplification of cDNA from *Bacteroides* spp with *wcfR*-specific primers to verify expression of the *wcfR* gene in the growth conditions employed. *B. intestinalis* is a negative control since its genome did not contain a homologue to *wcfR* in our genomic screen.

**Supplemental Fig. 2: Confirming disruption of one operon in each *B. cellulosilyticus* KO and *B. uniformis* KO.**

**A)** (**left panel**) Primers specific to the unique scaffolds and amplified cDNA confirmed that expression of ZPS1 was lost in *B. cellulosilyticus ΔZPS1* and ZPS2 was lost in *B. cellulosilyticus ΔZPS2*. (**right panel**) amplification of gDNA with a forward primer specific to *wcfR* and a reverse primer specific to the plasmid confirmed that pKNOCK-bla-Erm-gB had integrated into the *wcfR* gene in *B. cellulosylticus* KOs (gel cropped to put ladder adjacent to bands for figure). **B)** Confirmation of plasmid integration into the wcfR gene in *B. uniformis.*


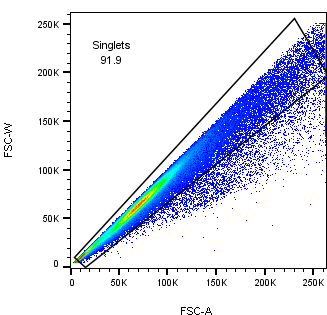

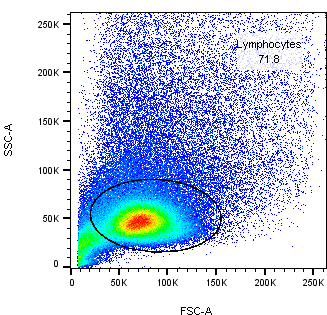

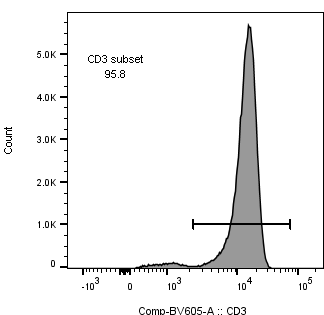

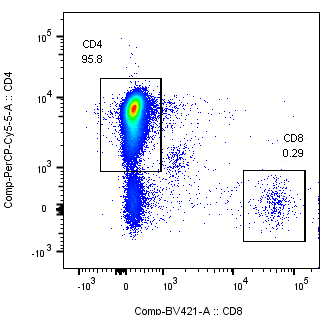

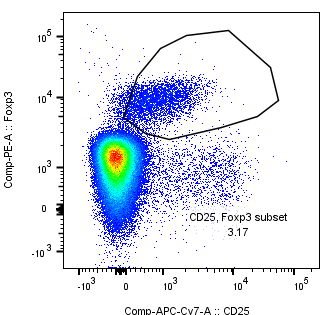

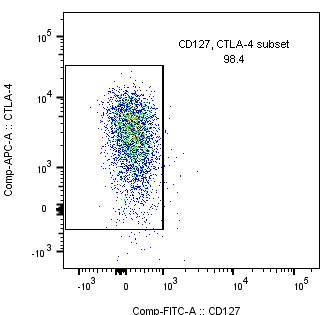


FSC-W

SSC-A

FSC-A

FSC-A

count

CD3

CD4

CD8

CD25

Foxp3

CTLA-4

CD127

**Supplemental Fig. 3: Gating strategy for CD25+FoxP3+CTLA4+CD127- cells of CD4+ T cells cultured for 3 days.**

Representative staining for CD25+FoxP3+CTLA4+CD127- Tregs in human PBMC and naïve T cells. Cells were stained for CD3, CD4, CD8, CD25, CD127, FoxP3 and CTLA-4.


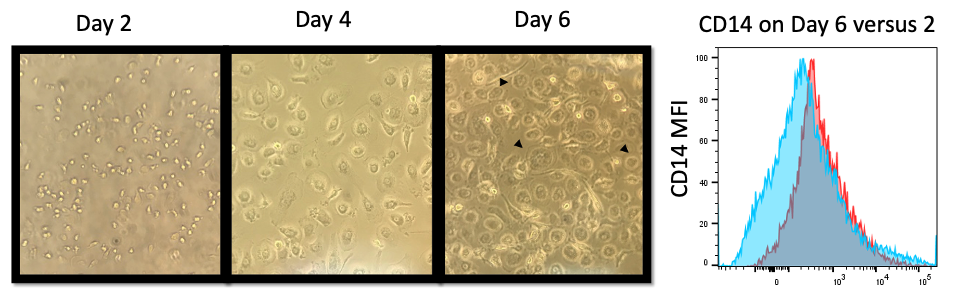


**Supplemental Fig. 4: Macrophage cultures derived from PBMC.**

**Left Panel:** Light microscopy of the process of differentiation of CD14+ cells purified out of PBMC. CD14+ cells were incubated at 37°C for 7 days with media containing 20 ng/ml macrophage colony stimulating factor (M-CSF). Arrows in third panel highlight characteristic macrophages. **Right panel:** CD14 MFI was evaluated using flow cytometry on Day 6 (blue) and Day 2 (red) to verify a reduction of this marker upon differentiation to macrophage.

**Supplemental Fig. 5: IL-6 and TNF-α levels from derived macrophages stimulated with *B. uniformis*, *B. uniformis* ΔwcfR or Zymosan.**

Macrophages were stimulated with heat-killed bacterial lysates for 6 hours, supernatant was collected, and ELISA was used to assess TNF-α and IL-6 levels. Statistical significance was assessed using the Friedman test.

| Target | Sequence |
| --- | --- |
| General *wcfR* | Forward: 5- GGR CGY ATC YTKGTG ATG AA -3  Reverse: 5- CCG ACA ATR TCA TAA CGC CA -3 |
| *B. cellulosilyticus* *wcfR* Scaffold 5 | Forward: 5- GAG TAA CAG CTG AAG ATG AAG TG -3  Reverse: 5-CTT CGG CCG TCG TCG TCA CAT TC -3 |
| *B. cellulosilyticus* *wcfR* Scaffold 9 | Forward: 5- GAG TAA CAG CTG AAG ATG AAG TG -3  Reverse: 5- AGT CGA AAG CTG GTG GGT GGC -3 |
| pKNOCK_ermGb | Forward 5- CGG GCT GCA GGA ATT CGA TA -3  Reverse 5- TTG CTG TTA ATG GCA GAG GTA -3 |
| *B. uniformis* insert | Forward 5-TGT GAA CTC GTG GAC ATC GG -3  Reverse 5- TGC GCT CAT AAT CGC AAG GA-3 |
| *B. cellulosilyticus* insert | Forward 5- ACG CGT CGA CTG CGG GTG CAA AGC CTG TTA TG -3  Reverse 5- CGG GGT ACC TCC TGT ACC AAT GAC GTC GC-3 |

**Supplemental Table 1.** Primers used to validate *wcfR* gene expression in wild-type strains, confirm plasmid integration in KO bacteria, and to amplify strain specific inserts to be ligated into suicide vector.
